# Abnormal cerebellar processing of the neck proprioceptive information drives dysfunctions in cervical dystonia

**DOI:** 10.1038/s41598-018-20510-1

**Published:** 2018-02-02

**Authors:** T. Popa, C. Hubsch, P. James, A. Richard, M. Russo, S. Pradeep, S. Krishan, E. Roze, S. Meunier, A. Kishore

**Affiliations:** 1 Inserm U 1127, CNRS UMR 7225, Sorbonne Universités, UPMC Univ Paris 06 UMR S 1127, Institut du Cerveau et de la Moelle épinière, ICM, Paris, France; 2 Department of Neurology, AP-HP, Hôpital de la Pitié-Salpêtrière, Paris, France; 3 Comprehensive Care Centre for Movement Disorders, Department of Neurology, Sree Chitra Tirunal Institute for Medical Sciences and Technology, Kerala, India; 4 Department of Neurosciences, University of Messina, Messina, Italy

## Abstract

The cerebellum can influence the responsiveness of the primary motor cortex (M1) to undergo spike timing-dependent plastic changes through a complex mechanism involving multiple relays in the cerebello-thalamo-cortical pathway. Previous TMS studies showed that cerebellar cortex excitation can block the increase in M1 excitability induced by a paired-associative stimulation (PAS), while cerebellar cortex inhibition would enhance it. Since cerebellum is known to be affected in many types of dystonia, this bidirectional modulation was assessed in 22 patients with cervical dystonia and 23 healthy controls. Exactly opposite effects were found in patients: cerebellar inhibition suppressed the effects of PAS, while cerebellar excitation enhanced them. Another experiment comparing healthy subjects maintaining the head straight with subjects maintaining the head turned as the patients found that turning the head is enough to invert the cerebellar modulation of M1 plasticity. A third control experiment in healthy subjects showed that proprioceptive perturbation of the sterno-cleido-mastoid muscle had the same effects as turning the head. We discuss these finding in the light of the recent model of a mesencephalic head integrator. We also suggest that abnormal cerebellar processing of the neck proprioceptive information drives dysfunctions of the integrator in cervical dystonia.

## Introduction

Primary dystonia is in all likelihood a developmental disorder of sensorimotor circuits, involving both the cortico-striato-pallido-thalamo-cortical and cerebello-thalamo-cortical pathways^1^. Primary cervical dystonia (CD) is a focal dystonia characterized by involuntary posturing of the head in any of the three axes.

The two pathophysiological features of dystonia, *i.e*. the abnormal propensity of the motor cortex to develop plasticity and the abnormal somatosensory processing are considered as part of a common endophenotype favoring dystonia yet not directly involved in generating abnormal dystonic movements. Indeed, the cortical plasticity of the motor cortex *per se* was abnormal within the hand representation in writer’s cramp^2,3^ and in CD^4,5^, irrespective of whether the hand representation mapped a dystonic or non-dystonic body part. Abnormalities of somatosensory processing were noted in the non-affected body parts of patients with unilateral dystonia, in patients with CD and blepharospasm, and also in their unaffected relatives^6–10^. In keeping with the reasoning that such abnormalities are endophenotypic markers but not directly linked to symptoms, deep brain stimulation of the *globus pallidus internus* in CD alleviates only the dystonic symptoms, but not the somatosensory processing in patients^11^.

When the dynamic relation between the cerebellum and M1 was explored either with double-pulses^12^ or with two consecutive repetitive transcranial magnetic stimulations^13^ in patients with writer’s cramp (one over the cerebellum followed by one over M1), no effective output was found from the cerebellum to M1 for the dystonic hand representation. This also seemed to play a direct role in generating dystonic symptoms^13^.

We reasoned that if the defective cerebellar modulation of M1 plasticity participates in the hand cramping in focal hand dystonia, the same modulation might be spared in cervical dystonia patients whose hands are normal. We investigated how modulation of the cerebellar output influences the plasticity of the hand representation in the motor cortex in cervical dystonia patients.

The cortical plasticity was investigated using a rapid, excitatory paired associative stimulation (PAS) protocol^14,15^, and the cerebellar output was modulated by decreasing or increasing cerebellar cortex excitability by means of repetitive transcranial magnetic stimulation (continuous (cTBS) or intermittent (iTBS) theta-burst protocols)^16^. Since the head of every patient was turned spontaneously throughout the experiment, the healthy controls were recorded either with the heads straight or voluntarily turned. To further disentangle the role of neck sensory inputs during head turn, we also recorded the healthy volunteers with the head straight during vibration of a neck rotator muscle.

## Results

### Cerebellar control in normal straight head versus dystonic position

**Figure 1:**
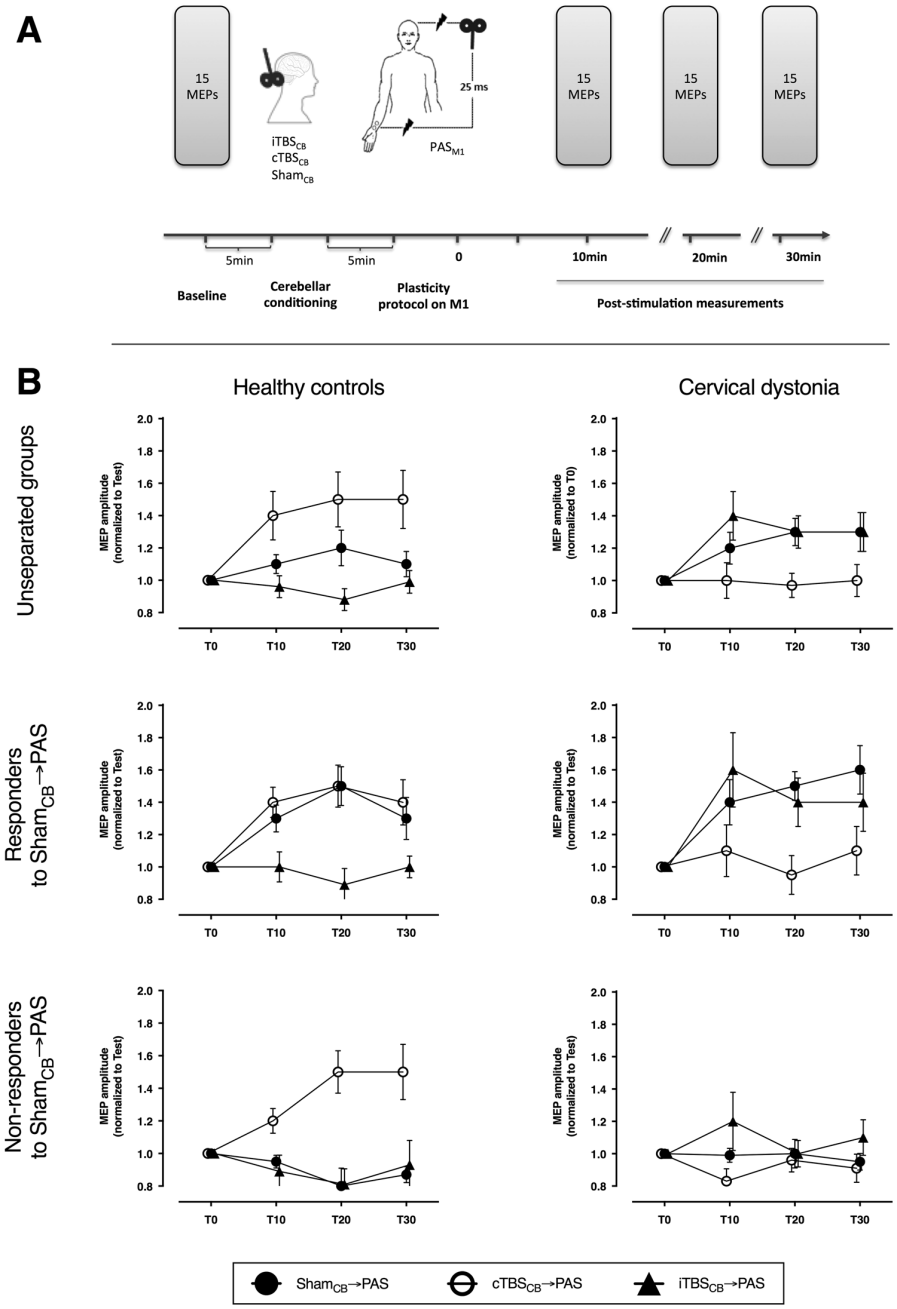
Experimental design and the comparison between healthy controls and patients with cervical dystonia. (**A**) Cortical excitability was quantified with the amplitude of motor potentials evoked with TMS pulses from the primary motor cortex, before and 10, 20, and 30 minutes after the repetitive stimulation. The repetitive stimulation consisted in real or sham stimulation of the cerebellum followed by PAS over M1 and median nerve. (**B**) The effect of the combined cerebellar and cortical stimulation in healthy controls and cervical dystonia patients are opposite. The effects are evident both when the groups are analyzed as a whole or split in responder and non-responders to PAS. cTBS_CB_: continuous theta-burst stimulation over the right cerebellar hemisphere; iTBS_CB_: intermittent theta-burst stimulation over the right cerebellar hemisphere; MEP: motor evoked potential; PAS: paired-associative stimulation; T0-T30: time points before the stimulation and at 10, 20, 30 minutes post-stimulation.

We compared two groups of subjects (one of patients with cervical dystonia (CD) manifesting as a right-sided torticollis and one of age- and gender-matched healthy controls, Table 1) in three different conditions (Fig. 1A) described before^16,17^: continuous theta-burst stimulation over the right cerebellum (cTBS_CB_), intermittent theta-burst stimulation over the right cerebellum (iTBS_CB_), and sham stimulation of the cerebellum (Sham_CB_), all three followed by a rapid paired-associative stimulation (PAS). Throughout these recordings, the CD did not constrain the position of their head, letting it rest in the dystonic position, while controls maintained the head in a neutral, straight position.

**Table 1:** Torticollis severity scores for the cervical dystonia patients (the scores were marked according to the TWSTRS).

CD patient	Age	Sex	Maximal Excursion						Duration factor	Effect of sensory tricks	Shoulder elevation	Range of motion	Time	Total
			Rotation	Laterocollis	Anterocollis	Retrocollis	Lateral shift	Sagittal shift						
1	66	M	0	1	0	0	1	0	10	1	2	2	3	**20**
2	49	F	1	0	0	0	0	1	4	1	0	0	2	**9**
3	43	F	3	0	0	0	0	0	8	2	0	2	4	**19**
4	64	M	1	0	0	1	0	1	2	0	0	1	0	**6**
5	39	F	1	1	1	0	0	0	8	1	0	2	4	**18**
6	47	F	1	2	0	0	0	0	6	1	0	2	3	**15**
7	57	F	2	0	0	0	0	1	6	1	1	2	4	**17**
8	33	M	1	0	0	0	0	0	2	2	0	1	1	**7**
9	64	F	1	2	0	0	0	0	4	1	1	1	4	**14**
10	62	F	1	1	0	0	0	0	8	1	0	1	3	**15**
11	50	F	2	1	0	0	0	0	8	1	0	0	3	**15**
12	37	M	3	2	1	0	0	0	10	1	3	4	4	**28**
13	45	F	2	1	1	0	0	0	10	1	2	3	3	**23**
14	22	F	2	1	0	0	0	0	4	1	0	2	2	**12**
15	24	M	0	2	0	0	1	0	8	1	1	1	2	**16**
16	48	F	2	1	1	0	0	0	8	1	3	4	4	**24**
17	47	M	1	1	0	3	0	0	10	1	1	3	4	**24**
18	59	M	4	1	0	0	0	0	10	0	0	3	4	**22**
19	55	F	1	0	0	3	0	0	8	0	0	1	3	**16**
20	54	M	4	0	0	0	0	1	2	1	0	3	4	**15**
21	61	M	2	1	0	0	0	0	6	0	1	2	0	**12**
22	62	M	0	0	0	0	1	0	8	0	1	3	3	**16**

The resting and active motor thresholds, and the size of the motor-evoked potentials (MEP) before any intervention did not differ between the two groups (Table 2). When the subjects were divided in only two groups, CD patients and controls (Fig. 1B), the ANOVA did not reveal any effect of individual factors (GROUP: CD and controls; INTERVENTION: Sham_CB_ → PAS, cTBS_CB_ → PAS, and iTBS_CB_ → PAS; TIME: T10, T20, T30), but one significant interaction (rmANOVA: GROUP: F_1,42_ = 0.01, P = 0.93; INTERVENTION: F_2,42_ = 1.2, P = 0.32; TIME: F_2,42_ = 0.1, P = 0.82; INTERVENTION * GROUP: F_2,84_ = 12.9, P < 0.0001). This suggests that the way in which the groups differ in their response to each intervention is more complex than what can be captured by this direct comparison.

**Table 2:** Physiological parameters for the 2 groups of healthy volunteers.

	RMT (% max stim output)	AMT (% max stim output)	Test MEP Sham_CB_ → PAS (mV)	Test MEP cTBS_CB_ → PAS (mV)	Test MEP iTBS_CB_ → PAS (mV)
Head straight N = 12	45.8 ± 7.2	39.0 ± 6.6	0.90 ± 0.27	0.88 ± 0.29	0.89 ± 0.22
Head turned to the right N = 12	46.2 ± 5.9	39.1 ± 7.2	1.04 ± 0.37	0.89 ± 0.35	1.25 ± 0.67

RMT: resting motor threshold, AMT: active motor threshold, MEP: motor evoked potential.

The basic response to PAS is known to be variable in the general population^18–20^. Following the previously validated approach to enhance the description accuracy of plastic phenomena^19,21^, we separated the subjects in responders and non-responders to PAS.

#### Responders to Sham_CB_ → PAS

The effects of the 3 interventions differed (Fig. 1B) between CD patients and the controls (rmANOVA: GROUP: F_1,24_ = 1.1 P = 0.3; INTERVENTION: F_2,48_ = 1.7, P = 0.02; TIME: F_2,48_ = 0.1, P = 0.9; INTERVENTION * GROUP: F_2,50_ = 58.7, P = 0.0006).

The post-hoc comparison of the INTERVENTION * GROUP interaction showed that the CD patients and the controls respond differently to each intervention. Unpaired t-test for Sham_CB_ → PAS revealed that the overall PAS effect in responder patients was significantly higher (CD vs controls: 1.53 ± 0.08 vs 1.33 ± 0.06, P = 0.049). This is in line with the previous reports that when a significant PAS effect is measured, it is stronger in dystonic patients^4,22^. Unpaired t-test for cTBS_CB_ → PAS (CD vs controls: 1.02 ± 0.08 vs 1.44 ± 0.07, P = 0.0003) and iTBS_CB_ → PAS (CD vs controls: 1.48 ± 0.1 vs 0.99 ± 0.05, P = 0.0001) revealed exact opposite responses in the two groups.

#### Non-responders to Sham_CB_ → PAS

The effects of the 3 interventions differed (Fig. 1B) between the CD patients and the controls, despite their “apparent” non-responsiveness to PAS (rmANOVA: GROUP: F_1,17_ = 1.2, P = 0.3; INTERVENTION: F_2,34_ = 4.4, P = 0.02; TIME: F_2,34_ = 0.4, P = 0.7; INTERVENTION * GROUP: F_2,34_ = 5.7, P = 0.007; INTERVENTION * TIME: F_4,68_ = 3.1, P = 0.02).

The post-hoc analysis of the INTERVENTION * GROUP interaction showed that the CD patients and the controls respond differently to each intervention. Unpaired t-test for Sham_CB_ → PAS revealed that the overall PAS effect was similarly low in both groups (CD vs controls: 0.97 ± 0.03 vs 0.88 ± 0.04, P = 0.1). This was expected, since both sub-groups were non-responders to PAS according to the selection criteria. Unpaired t-test for cTBS_CB_ → PAS (CD vs controls: 0.98 ± 0.06 vs 1.73 ± 0.2, P = 0.0008) and iTBS_CB_ → PAS (CD vs controls: 1.10 ± 0.07 vs 0.90 ± 0.06, P = 0.03) revealed exact opposite responses in the two groups. It is noteworthy that cTBS_CB_ can restore the PAS responsiveness in non-responder healthy controls, as previously described^19^.

The post-hoc analysis of the INTERVENTION * TIME interaction did not reveal any significant effect of TIME during any intervention (rmANOVA with TIME as repeated factor for Sham_CB_ → PAS: F_2,18_ = 0.5, P = 0.64; cTBS_CB_ → PAS: F_2,18_ = 3.3, P = 0.051; iTBS_CB_ → PAS: F_2,18_ = 1.9, P = 0.17), but a significant effect of INTERVENTION at the 2^nd^ and 3^rd^ time points, which was expected because the rTMS after-effects need ~10–15 min to reach their maximum level when they are not suppressed (paired t-tests at T10: Sham vs iTBS_CB_ P = 0.35, Sham vs cTBS_CB_ P = 0.17, iTBS_CB_ vs cTBS_CB_ P = 0.43; at T20: Sham vs iTBS_CB_ P = 0.70, Sham vs cTBS_CB_ P = 0.02, iTBS_CB_ versus cTBS_CB_ P = 0.02; at T30: Sham vs iTBS_CB_ P = 0.20, Sham vs cTBS_CB_ P = 0.02, iTBS_CB_ versus cTBS_CB_ P = 0.07).

### Cerebellar control in normal straight head versus normal, voluntary turned head position

In order to determine whether the newly identified differences in cerebellar modulation of motor cortex plasticity were specific to cervical dystonia or normally observed during head turning, we designed an additional experiment involving only PAS-responder, healthy subjects.

Two groups of 12 healthy subjects matched for age and gender underwent the same three interventions as in the previous experiment with the only difference being the different head-trunk alignment: one group kept the head in a neutral position facing forward throughout the stimulation and recording periods, while the other group maintained the head turned towards the right shoulder throughout the stimulation and recording periods.

The resting and active motor thresholds, and the MEP amplitudes before any intervention did not differ between the two groups (Table 2).

The effects of the 3 interventions differed according to the head position (POSITION: F_1,22_ = 0.5, P = 0.5; INTERVENTION: F_2,44_ = 0.5, P = 0.6; TIME: F_2,44_ = 4.7, P = 0.01; INTERVENTION*POSITION: F_2,44_ = 10.5, P = 0.0002; INTERVENTION * TIME * POSITION: F_4,88 = _2.5, P = 0.051).

**Figure 2:**
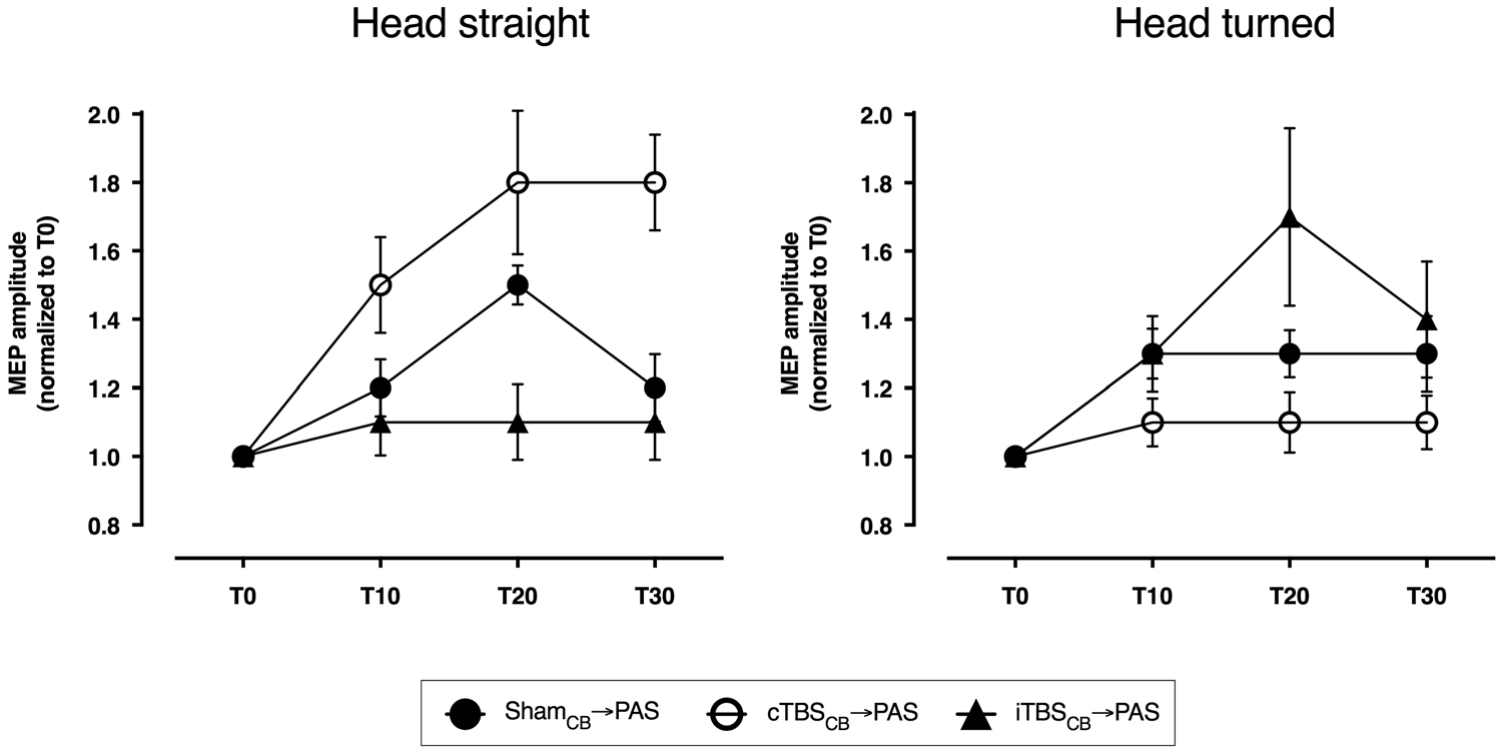
Cerebellar stimulation effects on motor cortex plasticity in healthy volunteers. Continuous theta-burst stimulation of the cerebellum enhances the PAS effect in responsive healthy subjects with the head straight, but blocks it when the head is turned. Intermittent theta-burst of the cerebellum has the exact opposite effect: it blocks the PAS effect in responsive healthy subjects with the head straight, but enhances it when the head is turned.

The post-hoc analysis of the INTERVENTION * POSITION interaction showed that the two head positions induce opposite results (Fig. 2), similar to what was observed between CD patients and controls. Unpaired t-test revealed that the overall effects of the Sham_CB_ → PAS did not differ between subjects maintaining the head turned and subjects maintaining the head straight (P = 0.67). Active cerebellar conditioning however, differed according to the head position for both iTBS_CB_ → PAS (P = 0.004) and cTBS_CB_ → PAS (P < 0.0001). The group maintaining the head straight reproduced the previous findings^16^, *i.e*. cTBS_CB_ significantly enhanced, while iTBS_CB_ prevented any plastic effect of PAS (paired t-tests Sham_CB_ → PAS vs cTBS_CB_ → PAS P = 0.003, Sham_CB_ → PAS vs iTBS_CB_ → PAS P = 0.005, iTBS_CB_  → PAS vs cTBS_CB_ → PAS P < 0.0001). In the group of subjects maintaining the head turned, the cerebellar effects were inverted, just as in the CD patients: cTBS_CB_ suppressed the PAS effects instead of enhancing it, while iTBS_CB_ only appears to enhance the PAS effects, though not significantly because of the high variability of the small cohort (paired t-test: Sham_CB_ → PAS vs cTBS_CB_ → PAS P = 0.006, Sham_CB_ → PAS vs iTBS_CB_ → PAS P = 0.2, iTBS_CB_  → PAS vs cTBS_CB_ → PAS P = 0.003).

### Cerebellar control in normal straight head position with and without vibration of neck muscles

In order to assess the role of neck proprioception in the inversion of cerebellar modulation of motor cortex plasticity, we have performed a control experiment on a small group of six healthy volunteers. They underwent vibration of the left sterno-cleido-mastoid (SCM) muscle while maintaining the head in the straight, neutral position. This was supposed to induce the illusion of a head rotation towards the contralateral side. In this control recording, we measured only the effect of cTBS_CB_ → PAS (as the paradigm with the biggest change). The results were compared to the cTBS_CB_ → PAS data recorded in age- and gender-matched healthy subjects from each of the responder groups in the experiment above (*i.e*., six maintaining the head straight and six maintaining the head turned).

**Figure 3:**
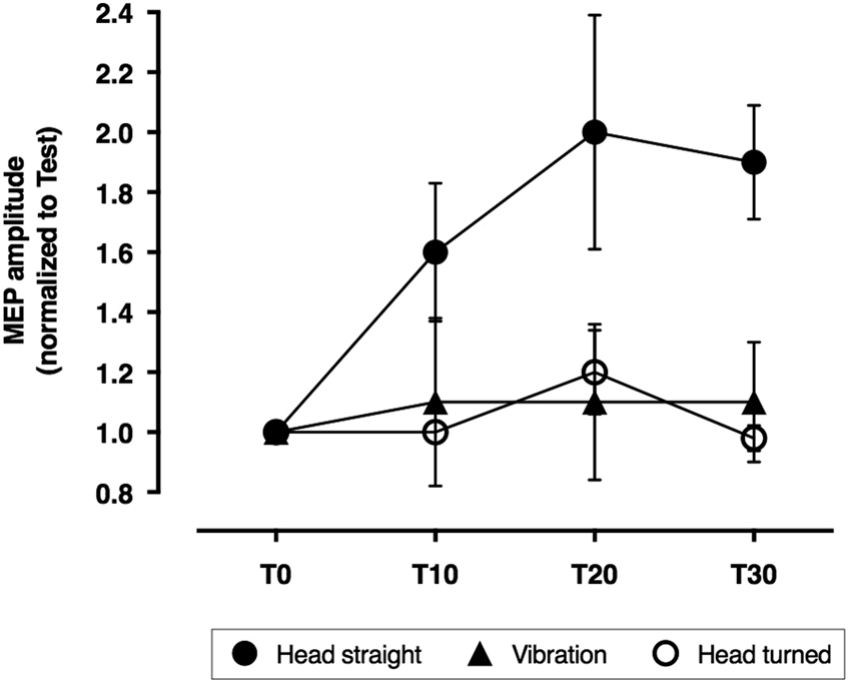
Control experiment on healthy volunteers. Continuous theta-burst stimulation applied over the right cerebellum enhanced the effect of PAS in responsive healthy subjects with the head kept straight, but blocked it in subjects with the head turned or undergoing vibration of the left sterno-cleido-mastroid muscle.

The cTBS_CB_ enhanced the responsiveness to PAS in the group with the head in the midline, but decreased it to the same extent in the other two groups (Fig. 3), the one maintaining the head turned and the one with the head in the midline but receiving SCM vibration (GROUP: F_2,15_ = 4.4, P = 0.03, TIME: F_2,30 = _2.8, P = 0.07; GROUP*TIME: F_4,30_ = 0.9, P = 0.5; post-hoc unpaired t-tests: head straight vs vibration P = 0.001, head straight vs head turned P < 0.0001, vibration vs head turned P = 0.78).

## Discussion

These results demonstrate for the first time that proprioceptive feedback from the neck plays a crucial role in the way in which the cerebellum can influence the plasticity of the primary motor cortex. We investigated the cerebellar influence over the M1 spike time-dependent plasticity in cervical dystonia patients with the head in a spontaneous dystonic position and age-matched healthy controls maintaining their heads either straight or voluntarily rotated to mimic the position of a right rotational torticollis. In healthy subjects maintaining their head straight in a neutral position, cTBS over the lateral cerebellum enhanced the contralateral M1 responsiveness to paired associative stimulation, while iTBS over the lateral cerebellum blocked it entirely, confirming previous reports^16,21^. When taking into account the individual responsiveness to PAS alone, these effects resulted more nuanced: in responders, cTBS_CB_ did not enhance the PAS effect significantly above the baseline response to PAS, while in non-responders, cTBS_CB_ did enhance it. The opposite was true for the iTBS_CB_: in responders, it effectively blocked the baseline response to PAS, while in non-responders, no significant effect could be observed since the PAS response was absent by default. Inversely, in CD patients, healthy controls maintaining the head rotated, and healthy controls maintaining the head straight during vibration of SCM, cTBS_CB_ and iTBS_CB_ had completely opposite effects on PAS: cTBS_CB_ blocked PAS effects and iTBS_CB_ enhanced them. The same nuances related to the responder/non-responder classification could be observed in CD patients, suggesting that the mirroring phenomenon is wide-reaching. This observation alone suggests that CD is a separate entity, in which the cerebellar output reaches and influences the motor circuits, unlike focal hand dystonia in which cerebellar output is virtually absent^12,13^.

The cerebellum is known to integrate multiple reference frames for motion by supporting a vestibular-somatosensory convergence^23,24^. However, most of the previous animal studies have concentrated on the multimodal integration of head-on-body and head-in-space with or without simultaneous whole-body rotation during an actual movement of the head. We have performed all recordings with the subjects maintaining a stationary position of the head either in their subjective “straight ahead” or in a voluntarily vertical rotation. This allowed us to exclude any confounding vestibular or fluctuating proprioceptive inputs. Changing the position of the head alone inverted the cerebellar effect on M1 plasticity but not the M1 plasticity *per se*, in all cases in which the head was rotated voluntarily, involuntarily, or illusorily.

The cerebellar TBS likely affects only the cerebellar cortex^25^ and particularly the horizontally orientated parallel fibers in the more superficial cerebellar molecular layer projecting on the Purkinje cells^26,27^. These parallel fiber-Purkinje cell synapses can undergo both postsynaptically expressed long-term depression or long-term potentiation, depending on whether or not the climbing fiber input is coactivated during tetanization, in which case the climbing fiber activity acts as a polarity switch^28^. Moreover, this differentiated plastic effect seems to coexist for different processes^29,30^. Considering that the molecular layer is where most of the multimodal inputs converge within the cerebellum^31,32^, we can infer that the neck proprioception alone might play a key role in the potentiation and the responsiveness of the parallel fiber-Purkinje cell synapses. A change in the responsiveness of these synapses might thus explain how the effects of cTBS_CB_ and iTBS_CB_ are inverted when the head is turned.

In order to minimize confounding factors, we have recruited only right-handed subjects and patients with predominantly right torticollis, and asked the healthy controls to maintain head rotation towards right. Despite mechanical differences between involuntary head rotations of the CD patients (manifested as complex movement, *i.e*. torticollis / laterocollis / anterocollis / retrocollis) and simple voluntary head rotation of the healthy controls, the direction and extent of the cerebellar priming of PAS effects were similar. This is in contrast with recordings in writer’s cramp patients, in whom both types of cerebellar stimulation did not influence PAS effects^13,33^.

In 2002, Klier and collaborators introduced the notion of a head and eye neural integrator^34^. They demonstrated in macaques that inactivation and stimulation of interstitial nucleus of Cajal (INC) cause head postures and oscillations resembling cervical dystonia: (1) left INC inactivation with muscimol resulted in right laterocollis and left torticollis, while right INC inactivation caused left laterocollis and right torticollis; (2) electrical excitation of INC resulted in head position changes exactly opposite of what were found with inactivation. This led to the idea that the postural symptoms of cervical dystonia in humans could be explained by severely imbalanced activity within such an integrator, either from intrinsic dysfunction or from an imbalance of inputs, or a combination of these two^34,35^. Shaikh and colleagues have further expanded this concept demonstrating in humans the existence of a leaky head neural integrator in the midbrain^36,37^. The authors brought compelling arguments that malfunctions in any of the inputs to this head neural integrator can lead to abnormalities resembling CD. This concept is in agreement with and represents a solid convergence point for most classical and recent models pointing to dysfunctions in the cerebellum, basal ganglia, or neck proprioception. Therefore, the new model also emphasizes that dystonia is a clinical syndrome but with heterogeneity in the underlying biological causes.

Our results show that, in healthy subjects, proprioceptive input from the neck can substantially change the way in which cerebellar output influences M1 plasticity. Both artificially triggering this input by vibration and physiologically triggering it by head rotation leads to inhibitory stimulation of the cerebellar cortex to prevent PAS plasticity in M1. The fact that cerebellar cTBS had the same effect in CD patients in their own neutral head position suggests that the cerebello-thalamo-cortical communication might exist in CD, unlike in focal hand dystonia, but “locked” in abnormal processing of proprioceptive inputs. This is in line with the finding that external visuo-motor and force-field adaptations, both involving cerebellum, are normal in CD patients^38^, but abnormal when estimation of one’s own body-schema is involved^39,40^. It is not clear though whether the changes of the parallel fibers-Purkinje cells synapses are intrinsic to the cerebellum (and thus part of the pathophysiology of the disease) or brought about by secondary inputs (from neck proprioceptors and/or basal ganglia via the pontine nuclei). This leaves open the possibility that cerebellar stimulations employed here (both cTBS and iTBS) elicit different plastic changes from those elicited in healthy volunteers.

For a long time, basal ganglia were considered as the main structure generating dystonic symptoms. Indeed, asymmetric local field potentials in the pallidum were recently reported, suggesting an asymmetry in pallidal outflow^41,42^. Given that the cerebellum and basal ganglia are strongly interconnected^43,44^ and that dysfunction in one can entrain dysfunction in the other^45^, a new question arises: is this asymmetric outflow part of the abnormal input only towards the mesencephalic neural integrator of the head or is it the result of a constantly abnormal outflow from the cerebellum towards the basal ganglia? In either case, a palliative treatment approach such as DBS targeting the GPi seems to give positive results in CD^46^.

Neck proprioception plays a crucial role in both limb coordination and representation of the body-scheme^47,48^. In healthy subjects, neck muscle vibration induces disparity between subjective perception and objective position of the body midline^36,49–51^. Subjects with chronic neck pain show consistent impairment of joint position sense^52^. Patients with cervical dystonia display significant kinematic abnormalities in non-dystonic segments, which can be corrected with botulinum toxin injections in the neck muscles^39^. In our study, vibration of the sternocleidomastoid muscle inverted the effect of the cerebellar cTBS on PAS in the same way head turning did, *i.e*. cancelling the PAS effect instead of amplifying it. The small but fast vibration cycles almost selectively induce a one-to-one train of action potentials in the primary endings of the large-diameter group Ia afferent fibers^53,54^, which would eventually induce the illusion of active contraction of the respective muscle^55^ and the subsequent rotational illusion of the head towards right in our study^56^. It is known that neck proprioception is relayed to the cerebellar anterior vermis in cats^57,58^, but the influence over the cerebellar output towards M1 hand area suggests that its weight throughout the cerebellum might be wider than anticipated. It is worth noting that CD patients had the same inversion of the cerebellar effects on M1 plasticity at small head rotation angles (TWSTRS score for rotation: 1.5 ± 1.2) as the healthy controls had at large rotations. This suggests that the cerebellar circuits in CD might be hyper-excitable, over-responding to proprioceptive information that could be dismissed as noise in healthy subjects.

Repetitive TMS (both inhibitory and excitatory) over cerebellum, M1, or premotor cortex was reported to improve dystonic symptoms in CD^22,59–62^, albeit not always^33^. Considering the apparently intact cerebello-cortical communication and the wide range of simulations resulting in positive clinical effect, it is possible that the non-invasive stimulations are acting through breaking a self-enforcing and/or self-sustaining chain of abnormal integration of proprioceptive feedback. It remains now to determine whether this cycle concerns only the priming of motor plasticity or other processes as well, like other aspects of the motor control, sensory computations, or even cognitive processes.

In summary, these data are compatible with the novel conceptual framework for the pathogenesis of cervical dystonia based on an abnormal dialogue between the mesencephalic head neural integrator and its inputs. Among these inputs, proprioception, the cerebellum, and basal ganglia were cited as key sources of feedback^1,36,40,63^. Our results suggest that a likely abnormal integration of the neck proprioceptive information at the cerebellar level subsequently drives an abnormal functioning of the integrator, which creates a head turn and feeds a vicious circle during the manifestation of the dystonic phenomenon.

This framework suggests that novel therapies for cervical dystonia such as chronic electrical stimulation of the cerebellum might be only palliative, while modulation of proprioception using vibration or electrocutaneous stimulation devices might be more suitable.

## Methods

### Procedures

The subjects were seated comfortably in an armchair, with both hands resting symmetrically on a pillow in their lap. The motor “hot-spot” for *Abductor pollicis brevis* (APB) muscle was identified in the left motor cortex and marked on the default brain reconstruction with the help of an MRI-based neuronavigation system (eXimia 2.2.0, Nextim Ltd in the French lab; BrainSight 2, Rogue Resolutions in the Indian lab). It allowed us to maintain the placement and tilt of the stimulator coil throughout each session and from one session to the next in the same subject. MEPs were recorded in the right APB with disposable Ag/AgCl surface electrodes in a muscle belly–tendon montage. Responses were amplified (1000x) and filtered (100–3000 Hz) with a Digitimer D360 amplifier (Digitimer Ltd, Welwyn Garden City, UK), then digitally transformed with 10 kHz sampling rate (CED Power 1401MkII, CED Ltd., Cambridge, UK) and stored offline for analysis (Signal 4.02, CED Ltd., Cambridge, UK). The EMG activity was continuously monitored to ensure muscle relaxation. Trials contaminated by EMG activity anywhere within 500 ms around each MEP were discarded from the offline analysis.

Each experiment started by calculating the APB resting (RMT) and active (AMT) motor thresholds according to the standard procedure^64^. This was done using both the Magstim 200 and the SuperRapid^2^ magnetic stimulators (Magstim Company, Whitland, Wales, UK).

### Cerebellar stimulation

Right cerebellar lobule VIII was chosen as target, since it is part of the sensorimotor network^65^ and readily accessible to TMS. Previous studies^16,25,66^ verified that this target identified on individual MRI scans corresponds to a surface landmark localized ~2 cm lower and 4 cm lateral to the inion. Continuous or intermittent theta-burst stimulations^67^ were delivered over the target with a 70 mm figure-of-eight coil held with the handle pointing upwards (so that the induced current had caudal-to-rostral orientation) and connected to a SuperRapid^2^ magnetic stimulator. For the sham stimulation (delivered with a cTBS pattern), the coil was moved vertically 5 cm below the cerebellar target^25^. For both active and sham stimulations, 600 pulses were delivered at 80% AMT_rapidstim_.

### Paired associative stimulation

We used a low intensity, high frequency PAS^14^. The electrical pulses were delivered over the right median nerve at the wrist at 2.5x the sensory threshold or just below the motor threshold, whichever was lower. Each electrical pulse was followed 25 ms later by a magnetic pulse delivered over the APB’s hotspot in the left M1 at 90% AMT_rapidstim_. Six hundred pairs of stimuli were delivered at 5 Hz through a 70 mm figure-of-eight coil connected to a SuperRapid^2^ magnetic stimulator.

### Cortico-spinal excitability assessment

Effects of PAS intervention on the cortico-spinal excitability were assessed with single-pulse TMS delivered at 130% RMT_magstim200_ at 0.2 Hz, through a 70-mm figure of-eight coil connected to a Magstim200 stimulator. The direction of the induced current was posterior-to-anterior. Fifteen MEPs were averaged prior to the intervention (T0), and at 10 min (T10), 20 min (T20), and 30 min (T30) after the end of the PAS.

### Vibration of the sternocleidomastoid muscle

A DC motor with an eccentric on the shaft (Brüel & Kjær, Nærum, Denmark) produced 7 N peak-to-peak vibrations at 80 Hz, with the amplitude < 6 mm. The vibrator was applied on the belly of the left sterno-cleido-mastoid muscle, 1 cm above its distal insertion on the clavicle. The vibrator was kept at the same spot throughout the experiment. After the session, subjects were asked specifically about head tilt or rotation illusions: nobody reported explicit sensations of head turning or tilting in response to the vibration. This is similar to the previous reports in which dorsal neck vibration rarely produced illusions of head movement^56^.

## Experimental design

All participants to this study gave their written informed consent prior to the study. The experimental procedures were in accord with the Declaration of Helsinki and approved by the following Ethics Committees: IEC at SCTIMST (India), CPP Ile-de-France VI (France), and.

### Experiment 1

Twenty-three healthy subjects and 22 CD patients were recruited from 3 centers, all of them attended three sessions. In all three sessions, 5Hz-PAS was used to induce plasticity in the dominant (left) M1. PAS was preceded by right cerebellar stimulation consisting of the following three, randomized interventions: (i) cerebellar cortex excitation [cerebellar-intermittent theta burst stimulation (iTBS_CB_ → PAS)], (ii) cerebellar cortex inhibition [cerebellar-continuous theta burst stimulation (cTBS_CB_ → PAS)], or (iii) sham stimulation of the cerebellum (Sham_CB_ → PAS). The three sessions were separated by intervals of at least 1 week. Five Hz PAS was applied 5 min after the end of cerebellar conditioning (Fig. 1A). Dystonia severity was assessed from videos recorded at the beginning of the first session. The video protocol was designed to score the Toronto Western Spasmodic Torticollis Rating Scale (TWSTRS) and was used by all three centers. All videos from the three centers were rated offline by the same movement disorders specialist (H.C.).

### Experiment 2

Experiment 1 revealed difference between the group of CD patients and the control group. Experiment 2 was designed to answer the question whether such differences are related to neck muscles activation in the CD group while neck muscles were at rest in the healthy subjects. Two groups of 12 healthy subjects each were compared (Group 1: mean age: 39.9 ± 10.2 years, range 30–66, 8 women; Group 2: mean age 39.7 ± 11.3 years, range 24–64, 8 women). Subjects of each group underwent the same three interventions as in experiment 1, the only difference being the different head-trunk alignments. Subjects of Group 1 looked forward throughout the stimulation and the recording periods in each session. Subjects of Group 2 maintained a voluntary head turn towards the right shoulder (angle 40–50°) throughout the stimulation and the recording periods in each session.

### Control experiment

This experiment was designed to assess, the role of neck afferents versus change in gaze direction or vestibular stimulation during voluntary head turn on the cerebellar priming. An additional group of 6 healthy volunteers was enrolled. These subjects were tested with cTBS_CB_ → PAS, while they received a vibration of the left sterno-cleido-mastoid muscle and maintained the head in the midline. We chose to use cTBS_CB_ → PAS as the effect of the head turn was more significant on cTBS_CB_ → PAS-induced effects than on iTBS_CB_ → PAS-induced effects (see Results). We then compared the effects of cTBS_CB_ → PAS in this group (n = 6, mean age 35.2 ± 10.9 years, range 24–56) with those obtained in 2 subgroups of 6 subjects matched one by one for age and sex and extracted from group 1 of experiment 2 *i.e* tested with the head on the midline (n = 6, mean age = 34.2 ± 8.5, range 25–50) and from group 2 of experiment 2 *i.e* while maintaining a head turn (n = 6, mean age = 35.3 ± 8.9, range 24–50).

The datasets generated during the current study are available from the corresponding author on reasonable request.
